# Design and Functionalization of a µPAD for the Enzymatic Determination of Nitrate in Urine

**DOI:** 10.3390/molecules26216355

**Published:** 2021-10-21

**Authors:** Francisca T. S. M. Ferreira, Raquel B. R. Mesquita, António O. S. S. Rangel

**Affiliations:** CBQF—Centro de Biotecnologia e Química Fina—Laboratório Associado, Escola Superior de Biotecnologia, Universidade Católica Portuguesa, Rua Diogo Botelho 1327, 4169-005 Porto, Portugal; fferreira@ucp.pt (F.T.S.M.F.); arangel@ucp.pt (A.O.S.S.R.)

**Keywords:** microfluidic paper-based device, nitrate reductase, hydrophilic membrane, Griess reaction, urine samples

## Abstract

In this work, the design of a microfluidic paper-based analytical device (μPAD) for the quantification of nitrate in urine samples was described. Nitrate monitoring is highly relevant due to its association to some diseases and health conditions. The nitrate determination was achieved by combining the selectivity of the nitrate reductase enzymatic reaction with the colorimetric detection of nitrite by the well-known Griess reagent. For the optimization of the nitrate determination μPAD, several variables associated with the design and construction of the device were studied. Furthermore, the interference of the urine matrix was evaluated, and stability studies were performed, under different conditions. The developed μPAD enabled us to obtain a limit of detection of 0.04 mM, a limit of quantification of 0.14 mM and a dynamic concentration range of 0.14–1.0 mM. The designed μPAD proved to be stable for 24 h when stored at room temperature in air or vacuum atmosphere, and 60 days when stored in vacuum at −20 °C. The accuracy of the nitrate μPAD measurements was confirmed by analyzing four certified samples (prepared in synthetic urine) and performing recovery studies using urine samples.

## 1. Introduction

Nitrite and nitrate are both nitrogen anions found in the human body either due to an endogenous process or through ingestion of water and food, such as vegetables and processed meats [1]. These anions have been often associated with cancer, especially nitrite, either from direct ingestion or the nitrate reduction by bacteria in the digestive system [2,3]. When nitrite reaches the acidic environment of the stomach, and is combined with amine or amide, it may form toxic and carcinogenic compounds such as nitrosamines and nitrosamides [2]. Furthermore, nitrite acts both as a substrate for the respiration of tumor cells and a signal molecule for their growth. Thus, although not directly leading to cancer, it has been reported to accelerate the proliferation and malignant transformation of cancer cells [4]. Additionally, when absorbed into the bloodstream, nitrite can also cause methemoglobinemia, especially in infants. In this condition, nitrite reacts with the iron in hemoglobin, irreversibly converting it into methemoglobin and blocking the transport of oxygen [2,5]. As the human body is a complex cooperation of interconnected systems, the contents of nitrate and nitrite in biological fluids such as blood, urine, and saliva reflect the transformation and metabolism of these ions in the entire body [4]. For example, although the presence of nitrate in the human urine is to be expected, the presence of nitrite usually implies the existence of bacterial infection, with the conversion of nitrate to nitrite [6]. Therefore, monitoring nitrate levels becomes essential for the prevention of diseases such as infections and cancer.

Nowadays, the diagnosis of most conditions is supported by the analysis of two main biological fluids, blood and urine. Urine may provide useful information as it contains several proteins and metabolites that serve as important biomarkers in analysis [7,8]. In addition, its collection is a painless noninvasive procedure, with a low risk of contamination or dispersion of contagious diseases, it does not require specialized medical personnel, and it can be performed in private areas with limited resources and more often than the blood collection [7].

According to the World Health Organization (WHO), even with the advances in technology, performing diagnosis and treatments on location, especially in the most secluded areas, is still a challenge. In the last few years, research has been developed toward new diagnostic and treatment devices and techniques that follow the “ASSURED” Guidelines provided by the WHO: Affordable, Sensitive, Specific, User-friendly, Rapid and robust, Equipment-free, and Deliverable to end-users [9]. One of the recently developed devices that tries to respond to this issue are the microfluidic paper-based analytical devices (µPADs). First triggered by Whitesides’ group in 2007 [10], the interest in these devices has thrived and expanded to areas from clinical diagnosis to environmental monitoring and food analysis in only a few years [11]. This is easily justified by their many intrinsic advantages, such as being simple, portable, affordable, disposable, and providing rapid measurements on location. Moreover, after being assembled, they do not require complex equipment or specialized personnel to handle it, which makes them an interesting tool for point-of-care analysis [2,12]. Although there are numerous reports of different µPAD fabrication techniques, the main concept lies in the use of paper as the hydrophilic platform for the reaction and a hydrophobic area that limits the reaction zone. The cellulose fibers in the paper provide microchannels where the capillary force produced by the interaction between cohesion and adhesion forces is responsible for the flow of analytes and reagents without the need of external driving devices [9,13,14]. The most common reported methods of µPAD fabrication include wax printing, photolithography, inject printing, paper folding, and plasma treatment [14,15]. Each has its own set of advantages and limitations, which is why the patterning technique should be carefully chosen according to the device objective [14]. Furthermore, µPADs can also be constructed in a two-dimensional or a three-dimensional structure. While the 2D-µPADs are simpler and rely on the lateral flow of fluids, the 3D-µPADs, constructed by stacking and folding, make use of both lateral and vertical flow, which allows the possibility of performing several chemical reactions in a specific order and with a higher control and flow speed [11,13,15].

The last important step in the use of µPADs for analytical determinations is the detection technique used to generate the readout [14]. Among the most reported are the colorimetric, electrochemical, and chemiluminescent. Colorimetric detection is one of the most widely used in the world due to the simple and straightforward signal capture. For quick qualitative measurement, results can be obtained by the naked eye, but if a rigorous quantitative measurement is required, the signal can be acquired by portable devices, such as cameras and scanners, and image processing software can be used for color intensity measurement [9,11,15].

The focus of this work was to develop a simple microfluidic paper-based analytical device capable of performing the nitrate determination in human urine samples. The nitrate conversion to nitrite was ensured by the enzymatic reaction involving nitrate reductase and the co-factor NADPH [16,17], and the detection of the resulting nitrite was performed by the Griess reagent [18]. Enzymes are biological catalysts that usually present a high selectivity and specificity, which is why it is considered an important technique in the analysis of numerous analytes [19]. To effectively use the enzymatic reaction in a microfluidic paper device, a strategy to delay the vertical flow to enable a significant extent of the enzymatic reaction was designed. This was achieved by incorporating a hydrophilic membrane layer, enabling the control of the vertical flow rate. The incorporation of a hydrophilic membrane also provided a physical separation between the sample insertion layer and the color reaction layer, enabling direct sample introduction, minimizing potential interferences, namely sample intrinsic color. Additionally, the limitations of a previously reported device [2] fabrication process, namely the placement of the zinc powder on paper, were also tackled.

## 2. Materials and Methods

### 2.1. Reagents and Solutions

The solutions were prepared with analytical-grade chemicals and Milli-Q water, resistivity > 18 MΩ/cm (Millipore, Merck KGaA, Darmstadt, Germany).

A standard stock solution of 10 mM of potassium nitrate (Fluka, Merck KGaA, Darmstadt, Germany) was prepared monthly by dissolving approximately 10 mg of previously dried solid (overnight at 100 °C) in 10 mL of water. The working nitrate standards were prepared daily from the stock solution in the range of 0.2–1.0 mM.

A phosphate-buffered solution was prepared by dissolving 1.16 g of K_2_HPO_4_·3H_2_O (Merck, Darmstadt, Germany) and 3.5 mg of ethylenediamine tetraacetic acid (EDTA) (Merck, Darmstadt, Germany) in 200 mL of water. The pH was adjusted to 7.4 and the solution stored in the refrigerator when not in use.

A 20 mM NADPH solution was prepared by dissolving 16.5 mg of NADPH (tetrasodium salt, 98%, Roche, Basel, Switzerland) in 1 mL of water and stored in the refrigerator.

The nitrate reductase enzyme stock solution (from *Aspergillus species*, Roche, Basel, Switzerland) was prepared by adding 2 mL of water to the 20 U in the flask. This solution was separated in 100 µL aliquots and stored in a freezer. The 1.5 U/mL nitrate reductase (NR) solution was prepared daily by dilution of the 10 U/mL stock solution.

The enzyme solution (ES) placed on the µPAD was prepared upon use by mixing 120 µL of NR 1.5 U/mL and 15 µL of NADPH 20 mM.

The modified Griess reagent was prepared monthly according to Teixeira et al. [18] by dissolving separately 0.4 g of sulfanilamide (Sigma-Aldrich, Merck KGaA, Darmstadt, Germany) in 2 mL of 5 M of orthophosphoric acid and 0.04 g of *N*-(1-naphthyl)-ethylenediamine dihydrochloride (N1NED) (Merck, Darmstadt, Germany) in water. These two homogenized solutions were then mixed, and the volume was completed to 20 mL to final concentrations of 20 g/L of sulfanilamide and 2 g/L of N1NED. This solution was stored in a dark bottle, shielded from the light.

### 2.2. Design of the Developed µPAD

**Figure 1:**
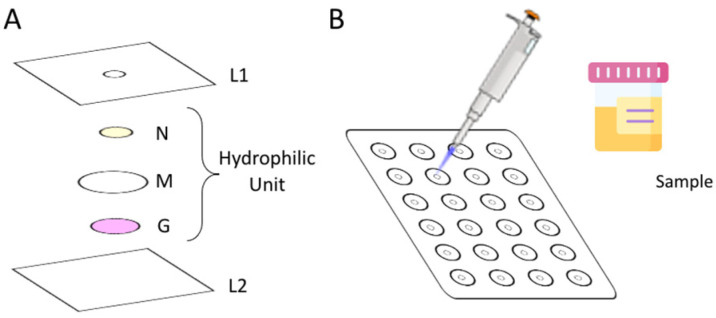
Schematic representation of: (**A**) the μPAD unit assembly and (**B**) sample insertion.

To assemble the µPAD, 24 hydrophilic units (Figure 1A) were aligned under the 3 mm holes (L1) previously perforated in a laminating pouch (75 × 110 mm, Q-connect), in a 4 column and 6 row distribution (Figure 1B). Each hydrophilic unit (Figure 1A) comprised 3 layers: the top layer, N, consists of a Whatman Grade 4 filter paper disc with 6.35 mm diameter loaded with enzymatic solution (ES); the middle layer, M, consists of a hydrophilic mixed cellulose ester membrane with 12.7 mm diameter (ME24 Whatman, Little Chalfont, Buckinghamshire, United Kingdom); and the bottom layer, G, consists of a Whatman Grade 50 filter paper disc with 5 mm diameter loaded with Griess reagent.

The ES disc from layer N was prepared by loading 5 µL of NR and NADPH mixture (enzymatic solution) in each disc and set to dry for 20 min in the oven at 37 °C.

The paper disc from the G layer was prepared by adding 10 μL of the Griess reagent to the discs and placing them in the oven to dry, for 10 min at 50 °C.

After the alignment of the units, the laminating pouches were passed through the laminator (United Office—ULG 300 B1, Cleveland, OH, USA), which forces the plastic pouch to melt and seal around the units, creating a strong physical barrier, the hydrophobic area. After the lamination, the μPADs were ready to be used.

The lamination is a delicate part of the assembly process, and a poor distribution of the units may result in a low reproducibility. Avoiding the shifting of the discs and units is essential; however, it can still happen, and, for that reason, it was established to have 8 units for one standard/sample (and collect the data of 3/4 replicates) to account for possible outliers.

### 2.3. Determination Procedure

To measure the concentration of nitrate in urine samples, 20 µL of standard/sample was placed at the insertion hole of the assembled µPAD (L1 layer in Figure 1), and then the sample/standard entered the µPAD and nitrate interacted with the nitrate reductase enzyme and NADPH (layer N in Figure 1), with nitrate being converted into nitrite. The hydrophilic membrane (layer M in the Figure 1) delays the flow through the sample and ensures that the sample is retained in the enzymatic layer for enough time to attain the nitrate reduction. After its complete absorption, the holes were covered with adhesive tape to prevent evaporation and possible contaminations. After the nitrate reduction, the formed nitrite passed through the membrane to the bottom layer with the Griess reagent (layer G in Figure 1), thus reacting and forming the pink color product. The intensity of the color was measured by scanning (Canon LiDE 120, Ōta, Tokyo, Japan) the bottom layer of the μPADs. The time lapsed between the sample/standard introduction and the scanning was set to 20 min and named time-to-scan (TTS).

The scanned images were processed using ImageJ (National Institutes of Health, Wisconsin, USA) by converting them into RGB plots. Then, the green filter was applied prior to measuring the intensity, as the expected colored product of the Griess reaction is pink (from which the complementary color is green). For each unit, a choice was made to perform the measurements using a circular selection of 200 × 200 pixels, as it allowed a better adjustment to the reagent disc area.

The intensity values were then converted into absorbance values using the formula: $A=I_{0}/I_{s}$, where $I_{0}$ is the intensity of the blank signal, obtained when loading with deionized water, and $I_{s}$ is the intensity of the standards/sample signal.

For each reading of the blank or standard/sample, 8 measurements were made, and outliers were removed when necessary. The remaining replicates were used for the average absorbance calculation. Then, the calculated absorbance values were plotted against the concentration of nitrate.

### 2.4. Samples Collection

The urine samples were collected as “blind samples” from volunteers with their informed consent and stored in the freezer at −20 °C until use.

### 2.5. Validation

To assess the accuracy of the μPAD measurements, a certified water sample was used, QC RW1 (VKI reference materials, Eurofins, Copenhagen, Denmark), as it consists of a concentrate preparation ampoule to be diluted. Therefore, different solutions were prepared in synthetic urine instead of water. The final nitrate concentrations were: 0.707 mM (AC1), 0.530 mM (AC2), 0.471 mM (AC3), and 0.354 mM (AC4).

For further validation assessment, recovery percentages were calculated based on the addition of 20/30 µL of 10 mM of nitrate standard to 1 mL of the urine samples.

## 3. Results and Discussion

As mentioned above, the color reaction chosen for the developed μPAD was the Griess reaction for nitrite determination, so the color reaction layer (layer G in Figure 1) was adapted from a previously reported work by Ferreira et al [2].

### 3.1. Preliminary Studies

To attain the nitrate determination based on the Griess color reaction for nitrite, it required the reduction of nitrate to nitrite, for which the enzymatic reaction using nitrate reductase was chosen. The enzymatic reaction, followed by the colorimetric reaction, was tested in a batchwise procedure based on the enzymatic assay protocol of Nitrate Reductase of SIGMA (EC 1.6.6.1).

#### 3.1.1. Incubation Period

According to the enzymatic protocol, there is an incubation step. To evaluate if this step could be avoided, the first study was to test the protocol with and without the incubation temperature. The studies were performed for nitrate concentrations of 4.3 and 2.8 mM, using both the incubation temperature reported in the protocol (30 °C) and room temperature (≈21 °C). As expected, the lower temperature produced a decrease in the efficiency of the enzymatic reaction, −31% for the 4.3 mM concentration and −23% for the 4.3 mM concentration. Nevertheless, because the incubation at 30 °C would impair the μPAD field application and, considering the target concentrations were below 2 mM, a choice was made to carry out the determination at room temperature.

#### 3.1.2. Enzymatic Reaction Conditions

To optimize the enzymatic reaction conditions, several combinations with different proportions of NR, NADPH, and nitrate were tested (Tables S1 and S2). The first set of studies (Table S1) were made using a nitrate standard of 12 mM, as recommended by the SIGMA protocol. Although this nitrate concentration was above the target range, these studies still enabled us to conclude that, for the same amount of NR, increasing the NAPDH amount did not cause a significant signal increase (Figure S1A). However, for the same amount of NADPH, the increase in NR amount resulted in an increase in the absorbance signal.

As the top expected nitrate concentration in urine sample was 1.0 mM, the previous studies were repeated using a nitrate concentration of 1.2 mM. The results showed that by increasing the NADPH amount, the absorbance slightly increased (Figure S1B). By increasing the NR amount, no significant variation in the absorbance was observed. In the end, the chosen conditions for 100 µL of a 1.2 mM standard corresponded to: 150 µL of 0.2 mM of NADPH and 100 µL of 0.4 U/mL of NR.

### 3.2. µPAD Design—Membrane Incorporation

For the µPAD design, a vertical flow was chosen to ensure the quantitative reduction prior to the colorimetric reaction, which means setting the first layer for the nitrate reduction. The nitrate in the sample would first have to be in contact with the NR and its cofactor, to enable the nitrate conversion to nitrite, which would then react with the Griess reagent layer to form a pink-colored product.

A strategy was devised for separating the two reactions and, accounting that the enzymatic reduction may need more time to proceed at sufficient extent, a choice was made to introduce an extra layer between the enzymatic reaction layer and the color reaction layer.

#### 3.2.1. Separation Layer

As mentioned, a strategy was designed to slow down the vertical flow by incorporating a membrane (hydrophilic membrane) between the enzymatic reaction layer and the color reaction layer. Then, the developed µPAD would be composed of three layers: the first layer, an impregnated paper with enzymatic solution (a mixture of NR and NADPH); the second layer, a hydrophilic membrane to increase the residence time in the first layer; and the third layer, an impregnated paper with Griess reagent for the colorimetric reaction. The enzymatic solution (ES) composition was based on the above-mentioned preliminary studies (Section 3.1).

#### 3.2.2. Membrane Selection

To increase the efficiency of the enzymatic reduction, it was essential to ensure enough reaction time, so a hydrophilic membrane was placed in the second layer. The size of the membrane disk was set to 12.7 mm to ensure complete separation between the enzymatic reduction and colorimetric reaction layers.

Using the same nitrate standard, different membranes were tested (Table 1) to assess which one produced the highest absorbance value. A higher value would mean that a higher amount of nitrite had reached the reagent layer, which would also mean that the membrane had caused a higher delay time in the first layer, thus promoting a higher reduction rate. The one that provided a higher absorbance signal was the CE20 membrane, so this was the chosen membrane for further studies.

### 3.3. µPAD Design—Enzymatic Reaction

After the physical assembly of the µPAD optimization, the concentrations of the NR and NADPH used in the enzymatic solution mixture were revisited and the sample volume studied.

#### 3.3.1. Paper Selection

**Figure 2:**
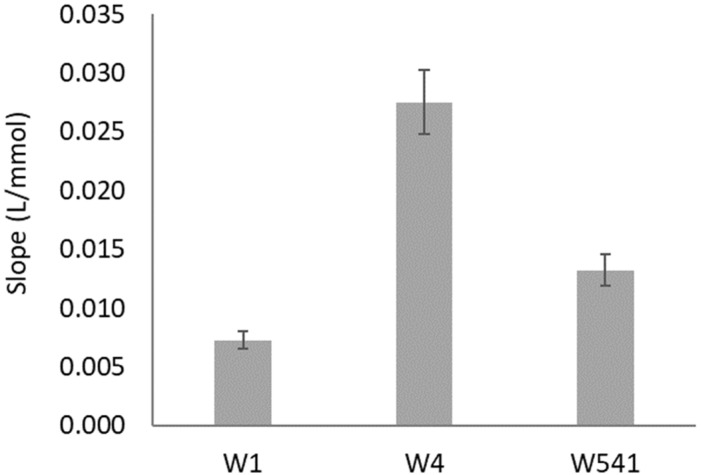
Study of filter paper type and porosity influence on the enzymatic reduction reaction.

The filter paper type and porosity used on the enzymatic reduction layer (layer N from Figure 1) were studied using two nitrate standards: 0.5 and 1.5 mM (Figure 2).

The most widely used and cheaper filter paper is the qualitative-grade Whatman 1 (W1), so that would always be the first choice. Then, Whatman 4 (W4) was tested, being of the same qualitative grade, but with higher porosity (Table S3) to facilitate the space arrangement of the enzymatic reaction. Maintaining the higher pore size, a hardened ashless grade paper (W541) was also tested to assess if the paper treatment would benefit the enzymatic reaction. The paper that provided a significantly higher sensitivity (slope) was W4 (Figure 2), so this was the paper used on the layer N for the remaining studies.

#### 3.3.2. Nitrate Reductase Concentration

**Figure 3:**
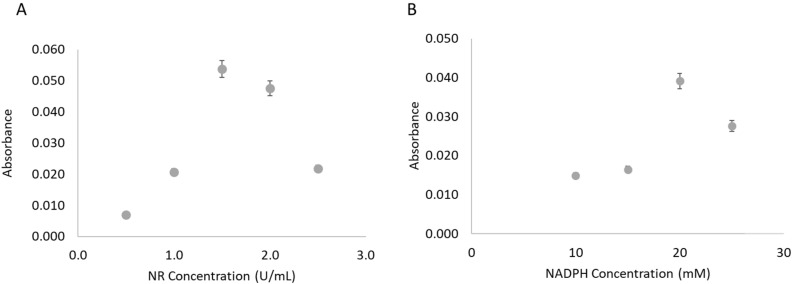
Study of the influence of the NR concentration (**A**) and NADPH concentration (**B**) on the enzymatic reduction reaction by calculation of the absorbance signal obtained for a 1.5 mM nitrate standard.

The NR concentration used in the enzyme solution mixture had been based upon preliminary studies, but it was revisited in the µPAD approach. The study was performed using the highest nitrate standard (1.5 mM) and in the range of 0.5–2.5 U/mL of NR (Figure 3A). The NR concentration that presented a higher value of absorbance, thus a higher reduction extent, was 1.5 U/mL of NR.

#### 3.3.3. NADPH Concentration

The influence of the NADPH concentration in the enzymatic solution was also studied using the 1.5 mM nitrate standard (Figure 3B). In the studied range, 10–25 mM, the concentration of 20 mM resulted in the highest absorbance value, inferring a higher conversion of nitrate to nitrite.

#### 3.3.4. Sample Volume

Trying to increase the sensitivity of the determination, a higher volume of sample, 25 µL, was tested. However, no complete absorption on paper occurred, not even after 1 h, which would not be compatible for a field application. This led us to believe that the maximum sample volume capacity of the µPAD had been reached with the 20 µL sample/standard, and so it was the chosen volume.

### 3.4. Features

The main characteristics of the developed μPAD including dynamic range, limit of detection (LOD) and quantification (LOQ), and relative standard deviation (RSD) are summarized in Table 2.

**Figure 4:**
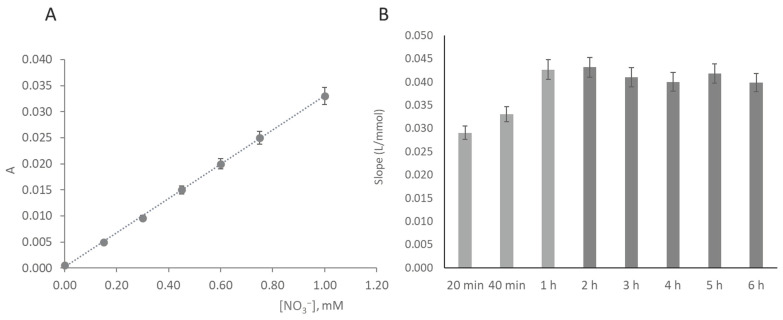
Calibration curve for the nitrate determination obtained with the developed µPAD: (**A**) calibration curve plotting; (**B**) calibration curve slope at increasing scanning times; light grey bars represent calibration curves with a correlation coefficient >0.99; dark grey bars represent calibration curves with a correlation coefficient <0.98.

The presented calibration curve was calculated as the average of 5 calibration curves (Figure 4A). The LOD and the LOQ were calculated as concentrations corresponding to three and ten times, respectively, the standard deviation of the intercept (n = 5), according to IUPAC recommendations [20].

The repeatability of the developed μPADs was evaluated by calculating the RSD of the calibration curve slope; two calibration curves in the same day for the intraday repeatability and four calibration curves in consecutive days for the interday repeatability.

#### 3.4.1. Scanning Time Interval

The enzymatic reaction requires time, and the chosen approach to incorporate a hydrophilic membrane intended to increase the residence time in the enzymatic layer. However, the idea was to have enough reaction extension and yet a minimal time between sample insertion and digital scanning, named time-to-scan (TTS), to ensure one of the μPADs’ most attractive characteristics, real-time analysis. For TTS assessment, a calibration curve was prepared, and the μPADs scanned at several time intervals after standard insertion, from 20 min up to 3 h (Figure 4B). As expected, the results showed that the sensitivity increased from the set TTS of 20 min to 40 min (14%) and up to 1 h (≈30%) as a consequence of more nitrite being formed and reaching the reagent layer. After 1 h, there was no further increase in the sensitivity and there was a significant decrease in linearity (Figure 4B), indicating that no more nitrite was reaching the bottom layer and that some degradation of the colored product might be occurring.

#### 3.4.2. Assessment of μPAD Stability

To evaluate the robustness of the developed μPAD, the stability of the developed device was tested when stored.

The μPADs were stored in plastic bags (Lacor, 69053, Bergara, Spain), under two different atmospheric conditions (air and vacuum), and at two temperatures, room temperature (approximately 21 °C) and in a freezer (approximately −20 °C). The vacuum atmosphere was obtained using a vacuum packaging machine (Henkovac—MINI/120-ST ECO, ‘s-Hertogenbosch, The Netherlands). All stored μPADs were shielded from the light. Different periods of time were assessed for each of the atmospheric conditions and temperature.

The sensitivity of the average calibration curves obtained from freshly prepared μPADs was then compared with the sensitivities obtained with μPADs under different storage conditions; then, a relative deviation (RD) below 10% was considered nonsignificant.

The calibration curves obtained with the devices stored for 24 h in air and vacuum at room temperature showed no significant difference when compared to the calibration curves average (RD < 7%). The calibration curves obtained with the devices stored for 60 days in a freezer and in a vacuum atmosphere still presented a similar sensitivity to the average of calibration curves (RD < 1%), which confirmed that the developed μPADs can be efficiently stored.

### 3.5. Interference Assessment

To assess potential interferences from the urine matrix, a set of μPADs were prepared to perform two calibration curves, one using standards prepared in water, and one using standards prepared in synthetic urine.

The synthetic urine was prepared according to Machado [21]: 10 g/L of urea, 0.07 g/L of uric acid, 0.8 g/L of creatinine, 5.2 g/L of sodium chloride, 0.1 g/L of lactic acid, 0.4 g/L of citric acid, 0.37 g/L of calcium chloride dihydrate, 0.49 g/L of magnesium sulphate heptahydrate, 1.41 g/L of sodium sulphate, 0.95 g/L of potassium dihydrogen phosphate, 1.2 g/L of potassium hydrogen phosphate, and 0.49 g/L of glucose.

There was no significant difference on the calibration curves slopes (relative deviation < 10%), thus indicating that no significant matrix interferences occurred. Therefore, to reduce the reagents consumption, the standards preparation in water was kept.

### 3.6. Application to Urine Samples—Validation

To evaluate the accuracy of the developed μPAD for nitrate measurements, four dilutions in synthetic urine of a certified sample were used (Table 3).

A linear relationship between the two sets of results was established: [NO_3_^−^]_μPAD_ = 1.03 (±0.30) × [NO_3_^−^]_CV_ − 0.022 (±0.163) where the values in brackets represent the 95% confidence interval (t-student analysis). As the slope and the intercept were not statistically different from 1 and 0, respectively, there was no statistical difference between the certified value and the μPAD measurement.

To further assess the accuracy, recovery studies were performed by spiking the samples with 20 μL and 30 μL of the nitrate standard stock solution of 10 mM in 1 mL of the urine sample. The calculation of the recovery percentages was made according to IUPAC [22]: Recovery = ([NO_3_^−^]_found_ − [NO_3_^−^]_initial_)/[NO_3_^−^]_added_; and they are listed in Table 4.

The average of the calculated recoveries was 99% with a standard deviation of 7%. A statistical test (*t*-test) was used to evaluate if the mean recovery value did significantly differ from 100%. For a 95% significance level, the calculated *t*-value was 0.25 with a correspondent critical value of 2.97.

The statistical results indicate the absence of multiplicative matrix interferences, indicating that the developed μPAD was applicable to urine samples.

#### Nitrite Testing

As mentioned above, the aim of the developed µPAD was to be applied to nitrate determination in urine samples, where nitrite is not supposed to be present. Nevertheless, nitrite presence testing can be incorporated if, in the same assembly, the top layer (N layer in Figure 1A) is loaded only with NADPH and no NR. In this case, the color product formed was an indicator of nitrite presence. If a quantification is intended, then a full calibration curve with nitrite standards and a µPAD without NR in the first layer would have to be established (Figure S2).

## 4. Conclusions

In this work, a novel microfluidic paper-based analytical device was developed for the quantification of nitrate in human urine samples. The use of the enzymatic reduction of nitrate, as opposed to using zinc powder [2], enabled a less laborious and easier-to-reproduce process of preparation and assembly of the device. Moreover, as far as we know, this is the first time the nitrate reductase enzyme has been used in a µPAD platform. To slow down the vertical flow and thus promote the reduction reaction, a hydrophilic membrane was placed below the enzymatic layer, which also enabled us to overcome potential interferences. Among these were the urine samples’ intrinsic color, which was no longer a problem facilitating its application. This innovative approach added significant advantages without compromising the analytical features and can be used in other analytical situations where these challenges occur. The previously reported µPADs for nitrate determination (Table 5) use either zinc or vanadium (III) as a reducing agent, which emphasizes the innovative approach of the developed µPAD.

Additionally, there was no application to urine samples, probably due to the problem of potential color interference, which was tackled in our work with the membrane approach.

Although a couple of works did present better LODs and LOQs, the dynamic range reported here was quite suitable for the determination in the target samples with a more economic and environmentally friendly fabrication process.

In terms of stability of the developed device before use, the µPAD showed to be stable for 24 h stored at room temperature in air or vacuum atmosphere, and for 60 days stored in vacuum at −20 °C.

The characteristics of the developed µPAD for nitrate determination in urine make it suitable for point-of-care analysis.

## Figures and Tables

**Table 1:** Tested hydrophilic membranes and their characteristics.

Membrane ID	Material	Porosity (µm)	Supplier
Ny45	Nylon	0.45	Whatman Nylon (7404-004)
CA45	Cellulose Acetate	0.45	Whatman OE67 (1040-4014)
CE20	Cellulose Ester	0.20	Whatman ME24 (1040-1714)
CN45	Cellulose Nitrate	0.45	Sartorius (11406)
CN20	Cellulose Nitrate	0.20	Sartorius (11407)
P20	Polyethersulfone	0.20	Gelman

**Table 2:** Features of the developed μPADs for the determination of nitrate; LOD, limit of detection; LOQ, limit of quantification; RSD, relative standard deviation.

Dynamic Range (mM)	Calibration Curve ^a^ A = S × [NO_3_^−^] + b	LOD ^a^ (mM)	LOQ ^a^ (mM)	Repeatability, RSD	
				Intraday ^b^	Interday ^c^
0.14–1.0	y = 2.7 × 10^−2^ (±4 × 10^−3^) × [NO_3_^−^] + 6 × 10^−4^ ( ±4 × 10^−4^)	0.04	0.14	8%	5%

^a^ n = 5; ^b^ n = 2; ^c^ n = 4.

**Table 3:** Analysis of certified samples performed with the nitrate μPAD; CI, confidence interval at 95%; SD, standard deviation; RD, relative deviation.

Certified Sample Dilution	Certified Value, [NO_3_^−^] ± CI [mM)	µPAD [NO_3_^−^] ± SD [mM)	RD (%)
AC1	0.707 ± 0.011	0.717 ± 0.056	1.0
AC2	0.530 ± 0.008	0.491 ± 0.030	−7.4
AC3	0.471 ± 0.007	0.483 ± 0.067	2.5
AC4	0.354 ± 0.006	0.346 ± 0.020	−2.1

**Table 4:** Recovery percentages studies; standard deviation (SD); relative standard deviation (RSD).

Sample ID	Initial			Added [NO_3_^−^] (mM)	Found			Recovery (%)
	[NO_3_^−^] ± SD (mM)		RSD (%)		[NO_3_^−^] ± SD (mM)		RSD (%)	
U1	0.79	0.05	6.6	0.300	1.09	0.06	5.9	100
U2	0.55	0.03	5.5	0.200	0.76	0.05	7.0	103
U3	0.23	0.03	15	0.200	0.44	0.03	7.2	101
U4	1.38	0.04	2.7	0.200	1.55	0.13	8.1	85
U5	<LOD			0.200	0.21	0.02	8.7	81
U6	0.096	0.011	11	0.200	0.29	0.03	12%	95
U7	0.316	0.016	5.1%	0.200	0.53	0.05	8.5	106

**Table 5:** Summary of the characteristics of the developed µPAD in comparison with previously described devices; LOD, limit of detection; LOQ, limit of quantification.

Sample	Dynamic Range (mM)	LOD (mM)	LOQ (mM)	Reduction Agent	Fabrication Method	Reference
Water	0.05–1	0.019	0.048	Zinc	Inkjet printer	[23]
Food products	0.16–0.81	0.058	0.19	Zinc	Screen-printing	[24]
Food products	0.008–0.64	0.006	0.023	Vanadium (III)	Screen-printing	[25]
Saliva	0.27–1.2	0.08	0.27	Zinc	Cutting and Lamination	[2]
Urine	0.14–1.0	0.04	0.14	Nitrate Reductase	Cutting and Lamination	This work

## Data Availability

Data is contained within the article or Supplementary Material.

## Supplementary Materials

The following are available online, Table S1: Description of the different parameters tested under each condition and the resulting absorbance signal: 100 µL of a nitrate standard of 12 mM (1.2 µmol); concentration of NADPH, 2 mM; concentration of NR, 0.4 U/mL, Table S2: Description of the different parameters tested under each condition and the resulting absorbance signal: 100 µL of a nitrate standard of 1.2 mM (0.12 µmol; concentration of NADPH, 0.2 mM; concentration of NR, 0.4 U/mL, Table S3: Summary of some properties of Whatman Filter Papers used, Figure S1: Plot of the studied conditions in ESI Table1 and Table S2 with: (A) a 12 mM nitrate standard; and (B) a 1.2 mM nitrate standard; the error bars represent 5% relative deviation, Figure S2: Nitrite calibration curve; the error bars represent the standard deviation.

## Sample Availability

Samples of the developed devices are available from the authors.
