# Supplementary material for: Design and Functionalization of a µPAD for the Enzymatic Determination of Nitrate in Urine
Source: Molecules. 2021 Oct 21;26(21):6355. doi: 10.3390/molecules26216355 (PMC8588010; doi:10.3390/molecules26216355)
Supplement: Supplementary file 1 [file molecules-26-06355-s001.zip › molecules-1399631-supplementary.pdf]

Electronic Supplementary Information

Design and functionalization of a µPAD for enzymatic determination of nitrate ions in urine

Francisca T. S. M. Ferreira, Raquel B. R. Mesquita, António O. S. S. Rangel

Universidade Católica Portuguesa, CBQF - Centro de Biotecnologia e Química Fina – Laboratório Associado, Escola Superior de Biotecnologia, Rua Diogo Botelho 1327, 4169-005, Porto, Portugal

Table S1. Description of the different parameters tested under each condition and the resulting absorbance signal: 100 µL of a nitrate standard of 12 mM (1.2 µmol); concentration of NADPH, 2 mM; concentration of NR, 0.4 U/mL.

Condition ID	*NADPH*		*NR*		*A*
	Vol. (µL)	µmol	Vol. (µL)	U	
1A	100	0.2	100	0.04	*0.572*
2A	50	0.1	100	0.04	*0.626*
3A	100	0.2	50	0.02	*0.238*
4A	50	0.1	50	0.02	*0.238*
5A	50	0.1	150	0.06	*0.634*
6A	50	0.1	200	0.08	*0.742*

Table S2. Description of the different parameters tested under each condition and the resulting absorbance signal: 100 µL of a nitrate standard of 1.2 mM (0.12 µmol; concentration of NADPH, 0.2 mM; concentration of NR, 0.4 U/mL.

Condition ID	*NADPH*		*NR*		*A*
	Vol. (µL)	µmol	Vol. (µL)	U	
1B	50	0.01	100	0.04	*0.101*
2B	50	0.01	150	0.06	*0.097*
3B	50	0.01	200	0.08	*0.091*
4B	100	0.02	100	0.04	*0.181*
5B	150	0.03	100	0.04	*0.275*
6B	200	0.04	100	0.04	*0.292*
7B	150	0.03	150	0.06	*0.270*
8B	150	0.03	200	0.08	*0.278*

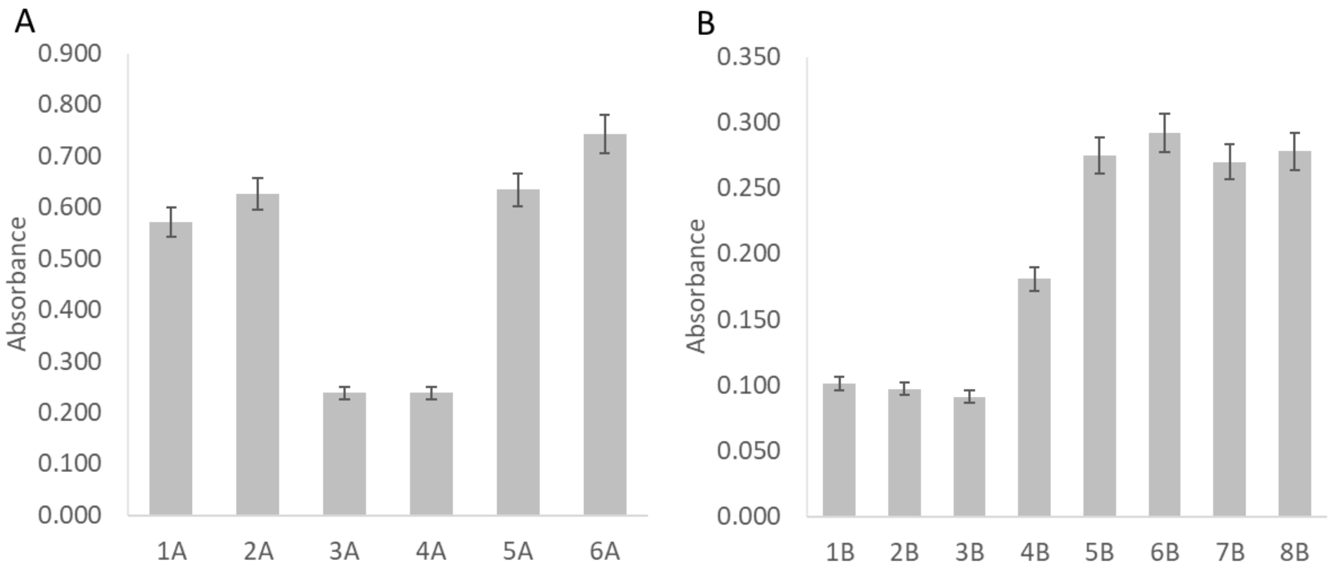

Figure S1. Plot of the studied conditions in ESI Table1 and Table S2 with: A) a 12 mM nitrate standard; and B) a 1.2 mM nitrate standard; the error bars represent 5% relative deviation.

Table S3. Summary of some properties of Whatman Filter Papers used

Treatment	*Paper*	*Particle Retention (μm)*
Qualitative	1	11
	4	20-25
Hardened Ashless	541	20-25

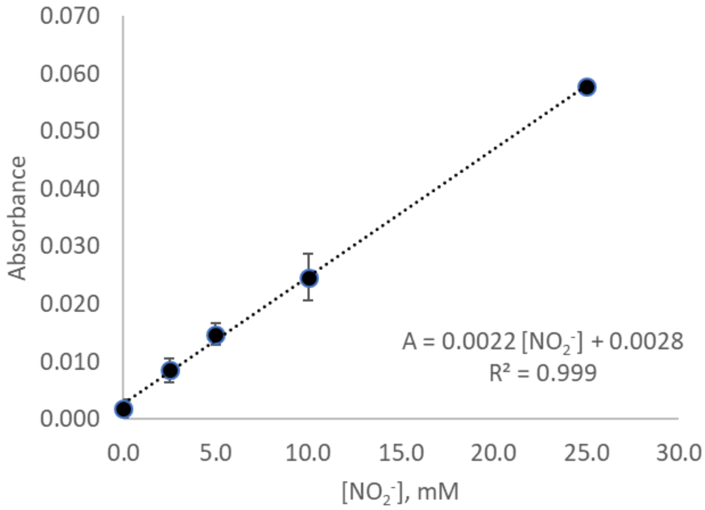

Figure S2. Nitrite calibration curve; the error bars represent the standard deviation.
